# Introduction of Breast Milk Substitutes During the First 3 Days of Life: Results of the Mexican National Survey of Demographic Dynamics, 2018

**DOI:** 10.1089/bfm.2023.0190

**Published:** 2024-01-17

**Authors:** Clara Luz Sampieri, Karina Gutiérrez-Fragoso

**Affiliations:** ^1^ Instituto de Salud Pública, Universidad Veracruzana, Xalapa, México.; ^2^ División de Ingeniería en Sistemas Computacionales, TecNM-Instituto Tecnológico Superior del Oriente del Estado de Hidalgo (ITESA), Apan, México.

**Keywords:** breastfeeding, breast milk substitutes, mother–baby pairs, Mexico

## Abstract

**Background:**

The introduction of foods or fluids other than breast milk in the first few days after birth interferes with the establishment of breastfeeding. This study aimed to investigate the association of formula introduction during the first 3 days of life with maternal sociodemographic characteristics, hospital practices, and breastfeeding duration.

**Materials and Methods:**

Information from the National Survey of Demographic Dynamics, 2018, which includes 17,686 mother–baby pairs was analyzed. Mother–baby pairs were classified into categories according to breastfeeding duration: <5 months and ≥5 months. Statistical methods and a machine learning algorithm (Bayesian network, BN) were used to analyze the data.

**Results:**

In general, 3,720 (21%) mothers reported introducing formula during the first 3 days of life. A lower education level, lower sociodemographic stratum, living in a rural area, and considering oneself indigenous were factors associated with not introducing formula during the first 3 days of life. A total of 5,168 (29.2%) mother–baby pairs practiced breastfeeding for <5 months, and 12,518 (70.8%) for ≥5 months. Almost twice as many mothers who practiced breastfeeding for <5 months introduced formula during the first 3 days of life (31.7%) compared with those who practiced breastfeeding for ≥5 months (16.6%). The BN model can sufficiently predict cases with a breastfeeding duration ≥5 months (precision–recall curve area = 0.792).

**Discussion:**

Introducing formula during the first 3 days of life was associated with a shorter breastfeeding duration. BN analysis showed a probabilistic dependency between the type of delivery and variables associated with the establishment of breastfeeding.

## Introduction

Breastfeeding has been proposed as a cost-effective public health intervention because the survival benefits span the entire continuum of childhood.^1^ The World Health Organization (WHO) recommends the initiation of breastfeeding within the first hour of life and then exclusively breastfeeding for the first 6 months of life, “meaning no other foods or liquids are provided, including water.”^2^ The WHO states that infants “should be breastfed on demand—that is as often as the child wants, day and night; no bottles, teats, or pacifiers should be used”; and that from the age of 6 months, they “should begin eating safe and adequate complementary foods while continuing to breastfeed for up to two years of age or beyond.”^2^ Early supplementation with infant formula has been associated with a shorter breastfeeding duration and decreased exclusive breastfeeding rates during the first 6 months of life.^3^ Exclusive breastfeeding under 6 months has been defined by the WHO and the United Nations Children's Fund (UNICEF) as the “percentage of infants 0–5 months of age who were fed exclusively with breast milk during the previous day.”^4^

In Mexico, the National Survey of Demographic Dynamics 2014 (known as ENADID with its Spanish acronym) estimated that 12.9% of infants were exclusively breastfed and that the mean breastfeeding duration was 9.8 months.^5^ The ENADID 2018 included questions about providing breast milk substitutes: infant formula, powdered milk, or bovine milk; water or tea; egg; atole, cereals, tortillas, or bread; juices or broths; and purees.^6^

Bayesian networks (BNs) are probabilistic models represented by directed acyclic graphs in which nodes represent variables and arcs represent dependent or independent relationships.^7^ BNs can be applied in classification, prediction, and diagnosis tasks.^8^ Although BN relationships are not necessarily causal, they show the influence of all analyzed variables.^9^ The objective of this study was to investigate the association of infant formula introduction during the first 3 days of life with maternal sociodemographic characteristics, hospital practices, and breastfeeding duration. To pursue this objective, statistical methods and BN models were used to analyze data from the ENADID 2018.

## Materials and Methods

### Study design

This was a descriptive study in which the data produced by the ENADID 2018 were analyzed.

### Ethical aspects

Ethics approval and signed informed consent are not applicable to this study because the data generated by the ENADID 2018 survey are considered of national interest according to the law of the National System of Statistical and Geographic Information in Mexico (known as SNIEG with its Spanish acronym).^10,11^ The SNIEG is coordinated by the Mexican National Institute of Statistics and Geography (known as INEGI with its Spanish acronym).^11^ Participants did not sign an informed consent form; the personal data of the participants are strictly confidential, and the participants were asked to provide the data in a timely matter and to ensure that their information was as accurate as possible. The database is public and is hosted on the INEGI's website, including the design of the methodology, validation of the instrument, and data collection. Subsequent analysis does not require approval.^6^

### The ENADID 2018 survey

The ENADID 2018 survey was designed, validated, applied, and analyzed by the INEGI. Public data from this survey are available for the years 1992, 1997, 2006, 2009, 2014, and 2018.^6^ The ENADID 2018 survey aims to provide statistical information about demographic dynamics, fertility, mortality, and migration, as well as other issues related to the population, households, and housing.^12^ This survey contains two questionnaires: one for households and another for mothers.^10^ The validation process of the information collected by the INEGI for the ENADID 2018 was carried out through several procedures, including field validation, primary validation, and automatic validation.^13^

### Participants

All women between 15 and 54 years of age with habitual residence in Mexico who reported having had their last pregnancy between January 2013 and the date of the ENADID 2018 interview and who had given birth to a live-born child were included.^6^ The national average nonresponse rate obtained in the field was 10.47%.^13^

### Setting and data collection

The ENADID 2018 survey collected data through direct interviews in the participants' homes in Mexico from August 13 to October 5, 2018 and had national coverage with its sample size of 119,800 households.^10^

### The ENADID 2018 database

The original ENADID 2018 database (*N* = 26,587 mother–baby pairs) was filtered using the question “Did you breastfeed?” (*N* = 24,428). Subsequently, data were filtered according to the question “How many days or months old was the infant when he or she started receiving infant formula, powdered milk, cow's milk, etc.?” The possible answers were “less than one day,” “one day,” “two days,” or “three days.” “Less than one day” and “one day” were recategorized as “first day of life” (*N* = 22,780).

Mother–baby pairs were classified into two groups: those with a breastfeeding duration of <5 months (Group A) and those with a duration of ≥5 months (Group B); these groups were defined according to participants' answers to the following question: “For how long did you give (infant) breast milk or breastfeed?” We excluded 69 mother–baby pairs from this study due to a lack of data about their breastfeeding duration as well as 5,000 mother–baby pairs who were still breastfeeding and 25 who did not provide data about the location where their delivery took place. A total of 17,686 mother–baby pairs were included in the dataset for this analysis. In this study, we will refer to “infant formula, powdered milk, cow's milk, etc.” as formula.

### The ENADID 2018 variables

The variables analyzed from the ENADID 2018 survey were education level (less than or equal to secondary versus higher than secondary), disability or limitation (present versus absent), locality (rural versus urban), sociodemographic stratum (low and low–medium versus medium–high and high), whether the participants consider themselves indigenous (yes versus no), age (15–19 and 40–54 years versus 20–39 years), the type of delivery (cesarean section versus vaginal birth), delivery in a medical unit (yes versus no), skin-to-skin contact immediately after birth (no versus yes), and receiving an explanation of breastfeeding after delivery (no versus yes). Disability or limitation was delimited using two questions: “How many people normally live in this house, including children, elderly individuals, and people with disability?” and “Tell me the names of the members of your household, starting with the head of house. Include young children, elderly individuals, and people with disabilities.” The rest of the questions and analyzed variables have been previously described.^14^

Breastfeeding duration in days was calculated through multiplying the number of months by 30 or the number of years by 365; for participants with a breastfeeding duration of ≤23 hours, a value of zero was assigned.

### Statistical and BN analysis

Categorical variables are summarized using absolute frequencies and percentages. Continuous variables are expressed as medians and the interquartile range for nonnormal distributions. Proportions were compared using the chi-square test. Associations were determined using odds ratios (ORs). A *p* ≤ 0.05 was considered statistically significant. StatCal: Statistical Calculators Epi Info version 7.2.5.0, Sigma Stat 3.5 and Weka version 3.8.6 were used for analysis.

For machine learning analysis, predictions of the breastfeeding duration were analyzed through a binary classification: <5 months (Group A) and ≥5 months (Group B).^4^ The confusion matrix that was analyzed included the following: correctly classified cases in Group A (true positive, TP), correctly classified cases in Group B (true negative, TN), and misclassified cases in both groups (false positive, FP and false negative, FN). The metrics that were analyzed were the TP rate (sensitivity), FP rate, precision, recall, F-measure, area under the receiver operating characteristic curve (ROC area), and area under the precision–recall curve (PRC area). The ROC area was used as a measurement to characterize the tradeoff between the TP rate and FP rate. PRC area was used as a measure to depict the tradeoff between recall and precision.^15^ The following values were considered optimal and nonoptimal: TP rate (1.0, 0.0), FP rate (0.0, 1.0), precision (1.0, 0.0), recall (1.0, 0.0), F-measure (1.0, 0.0), ROC area (0.9, 0.5), and PRC area (1.0, 0.5).^16^

During machine learning analysis, some metrics can be affected when using unbalanced datasets, so a cost-sensitive approach was applied to overcome the difference in the size of the groups.^17^ A penalty of 2 was set on the cost matrix for misclassifications of Group A.

## Results

Information from a public database, including 17,686 mother–baby pairs was analyzed. A total of 5,168 (29.2%) mother–baby pairs reported having practiced breastfeeding for <5 months (Group A), and 12,518 (70.8%) reported having practiced breastfeeding for ≥5 months (Group B). There were differences between the median (P25, P75) age of the mothers in Group A (28 [23, 33]) and those in Group B (29 [24, 34], *p* < 0.001). In general, 3,720 (21%) mother–baby pairs reported introducing formula within the first 3 days of life. Differences in median breastfeeding duration in days were observed between mother–baby pairs that introduced formula within the first 3 days of life (180 [60, 360]) and those did not (270 [120, 360], *p* ≤ 0.001).

The characteristics of the groups are presented in Table 1. Most of the participants were in the low and medium–low sociodemographic stratum, lived in an urban locality, did not report a disability or limitation, did not consider themselves indigenous and delivered in a medical unit (Table 1). In Group A, there were differences in the proportions of most of the variables analyzed, except for disability or limitation, considering oneself indigenous and delivery in a medical unit (Table 1). In Group B, no differences were found in the proportions of disability or limitation, sociodemographic stratum, considering oneself indigenous and age (Table 1).

**Table 1.** Characteristics of Mother–Baby Pairs from the Mexican National Survey of Demographic Dynamics, 2018

	Breastfeeding duration					
Characteristic	Group A <5 months			Group B ≥ 5 months		
	Introduction of formula within the first 3 days of life					
	Yes* N* = 1,635	No* N* = 3,533	*p*	Yes* N* = 2,085	No* N* = 10,433	*p*
	*n *(%)	*n *(%)		*n *(%)	*n *(%)	
Education level						
Less than or equal to secondary	681 (41.7)	1,664 (47.1)	0.0001	1,017 (48.8)	5,909 (56.6)	0.0001
Higher than secondary	954 (58.3)	1,869 (52.9)		1,068 (51.2)	4,524 (43.4)	
Condition of disability or limitation						
Present	200 (12.2)	398 (11.3)	0.1563	230 (11.0)	1,154 (11.1)	0.4868
Absent	1,435 (87.8)	3,135 (88.7)		1,855 (89.0)	9,279 (88.9)	
Locality						
Rural	305 (18.7)	750 (21.2)	0.0160	577 (27.7)	3,224 (30.9)	0.001
Urban	1,330 (81.3)	2,783 (78.8)		1,508 (72.3)	7,209 (69.1)	
Sociodemographic stratum						
Low and low-medium	1,093 (66.9)	2,522 (71.4)	0.0005	1,657 (79.5)	8,294 (79.5)	0.488
Medium-high and high	542 (33.1)	1,011 (28.6)		428 (20.5)	2,139 (20.5)	
Considering oneself indigenous						
Yes	472 (28.9)	1,079 (30.5)	0.1112	802 (38.5)	4,177 (40.0)	0.0903
No	1,163 (71.1)	2,454 (69.5)		1,283 (61.5)	6,256 (60.0)	
Age groups, years						
15–19 and 40–54	274 (16.8)	468 (13.2)	0.0004	265 (12.7)	1,267 (12.1)	0.2351
20–39	1,361 (83.2)	3,065 (86.8)		1,820 (87.3)	9,166 (87.9)	
Type of delivery						
Cesarean section	962 (58.8)	1,725 (48.8)	0.0001	1,076 (51.6)	4,375 (41.9)	0.0001
Vaginal birth	673 (41.2)	1,808 (51.2)		1,009 (48.4)	6,058 (58.1)	
Delivery in a medical unit						
Yes	1,604 (98.1)	3,458 (97.9)	0.3005	2,033 (97.5)	10,087 (96.7)	0.0230
No	31 (1.9)	75 (2.1)		52 (2.5)	346 (3.3)	
Skin-to-skin contact immediately after birth						
No	392 (24.0)	666 (18.9)	0.0001	494 (23.7)	1,795 (17.2)	0.0001
Yes	1,243 (76.0)	2,867 (81.1)		1,591 (76.3)	8,638 (82.8)	
Receiving an explanation of breastfeeding after delivery						
No	266 (16.3)	433 (12.3)	0.0001	361 (17.3)	1,349 (12.9)	0.0001
Yes	1,369 (83.7)	3,100 (87.7)		1,724 (82.7)	9,084 (87.1)	

*n*, number of participants presenting the characteristics of interest. The percentage corresponds to the column.

In general, according to ENADID 2018 data, having a level of education less than secondary (OR 0.70, 95% confidence interval [CI] 0.65–0.76), living in a rural area (OR 0.78, 95% CI 0.71–0.84), belonging to a low or low–medium sociodemographic stratum (OR 0.82, 95% CI 0.75–0.89), and considering oneself indigenous (OR 0.86, 95% CI 0.80–0.93) were factors associated with not introducing formula during the first 3 days of life. On the other hand, age between 15 and 19 years or 40 and 54 years (OR 1.19, 95% CI 1.07–1.32), having a cesarean section (OR 1.56, 95% CI 1.45–1.68), delivering in a medical unit (OR 1.36, 95% CI 1.07–1.72), not having skin-to-skin contact immediately after birth (OR 1.46, 95% CI 1.33–1.59), and not receiving an explanation of breastfeeding after delivery (OR 1.38, 95% CI 1.25–1.53) were factors associated with introducing formula during the first 3 days of life. Disability or limitation was not related to introducing formula within the first 3 days of life (OR 1.04, 95% CI 0.93–1.17).

A higher percentage of mothers introducing formula within 3 days of life occurred in Group A, with 1,635 (31.7%), than in Group B, with 2,085 (16.6%), *p* = 0.0001. There were differences in the proportions of mothers who introduced breast milk substitutes between the groups (Table 2). Among mothers who introduced breast milk substitutes during the critical window of the first 3 days of life, most implemented this on the first day of life (Table 2). In both groups, there were no differences in the proportion of mothers who introduced formula within 3 days of life between those who did or did not deliver in a medical unit (Table 3).

**Table 2.** Introducing Breast Milk Substitutes During the First 3 Days of life. Data from the Mexican National Survey of Demographic Dynamics, 2018, *N* = 17,686

Breast milk substitute	Breastfeeding duration		
	Group A <5 months *N* = 5,168	Group B ≥ 5 months *N* = 12,518	*p*
	*n *(%)	*n *(%)	
Formula			
No introduction on first to third day of life	3,533 (68.4)	10,433 (83.3)	0.0001
Introduction on first day of life	1,233 (23.9)	1,621 (12.9)	
Introduction on second day of life	236 (4.6)	286 (2.3)	
Introduction on third day of life	166 (3.2)	178 (1.4)	
Water or tea			
No introduction on first to third day of life	4,691 (90.8)	11,682 (93.3)	0.0001
Introduction on first day of life	280 (5.4)	354 (2.8)	
Introduction on second day of life	103 (2.0)	219 (1.7)	
Introduction on third day of life	94 (1.8)	263 (2.1)	
Egg			
No introduction on first to third day of life	5,028 (97.3)	12,432 (99.3)	0.0001
Introduction on first day of life	140 (2.7)	83 (0.7)	
Introduction on second day of life	0 (0.0)	2 (0.0)	
Introduction on third day of life	0 (0.0)	1 (0.0)	
Atole, cereals, tortillas, or bread			
No introduction on first to third day of life	5,039 (97.5)	12,459 (99.5)	0.0001
Introduction on first day of life	124 (2.4)	55 (0.4)	
Introduction on second day of life	3 (0.1)	1 (0.0)	
Introduction on third day of life	2 (0.0)	3 (0.0)	
Juices or broths			
No introduction on first to third day of life	5,042 (97.6)	12,485 (99.7)	0.0001
Introduction on first day of life	119 (2.3)	21 (0.2)	
Introduction on second day of life	3 (0.1)	3 (0.0)	
Introduction on third day of life	4 (0.1)	9 (0.1)	
Purees			
No introduction on first to third day of life	5,045 (97.6)	12,442 (99.4)	0.0001
Introduction on first day of life	116 (2.2)	73 (0.6)	
Introduction on second day of life	1 (0.0)	0 (0.0)	
Introduction on third day of life	6 (0.1)	3 (0.0)	

*n*, number of participants who did or did not introduce breast milk substitutes. The percentage corresponds to the column.

**Table 3.** Introduction of Formula During the First 3 Days of Life Based on Delivery in a Medical Unit. Data from the Mexican National Survey of Demographic Dynamics, 2018, *N* = 17,686

Formula	Breastfeeding duration					
	Group A <5 months			Group B ≥ 5 months		
	Delivery in a medical unit					
	Yes* N* = 5,062	No* N* = 106		Yes* N* = 12,120	No* N* = 398	
	*n *(%)	*n *(%)	*p*	*n *(%)	*n *(%)	*p*
No introduction on first to third day of life	3,458 (68.3)	75 (70.8)	0.9699	10,087 (83.2)	346 (86.9)	0.0615
Introduction on first day of life	1,205 (23.8)	28 (26.4)		1,580 (13.0)	41 (10.3)	
Introduction on second day of life	234 (4.6)	2 (1.9)		280 (2.3)	6 (1.5)	
Introduction on third day of life	165 (3.3)	1 (0.9)		173 (1.4)	5 (1.3)	

*n*, number of participants who did or did not introduce formula. The percentage corresponds to the column.

According to the BN model, the unbalanced analysis correctly predicted 576 (11.1%) cases from Group A, and 4,592 (88.8%) were misclassified; in Group B, 12,014 (95.9%) cases were correctly predicted, and 504 (4.1%) were misclassified. Meanwhile, the cost-sensitive analysis correctly predicted 2,091 (40.4%) cases from Group A, and 3,077 (59.6%) were misclassified; in Group B, 9,634 cases (77.0%) were correctly predicted, and 2,884 (23.0%) were misclassified.

**FIG. 1.**
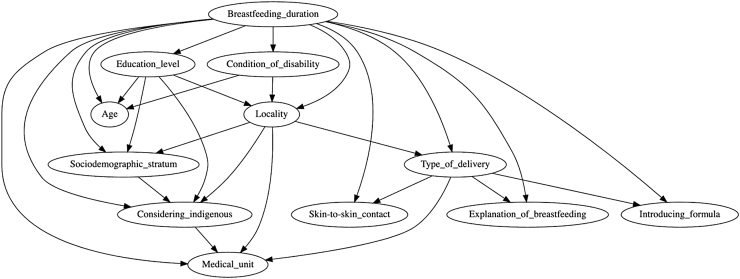
Bayesian network model of breastfeeding factors, sociodemographic and clinical practice variables. Data from the Mexican National Survey of Demographic Dynamics 2018, *N* = 17,686.

In both the unbalanced and cost-sensitive analyses, the type of delivery was associated with introducing formula within the first 3 days of life, with factors, including delivering in a medical unit, skin-to-skin contact immediately after birth and receiving an explanation about breastfeeding after delivery (Fig. 1). Both approaches also showed a probabilistic relationship of dependency between educational level, locality, sociodemographic stratum, considering oneself indigenous and delivering in a medical facility (Fig. 1). Except for the ROC and PRC area values, metrics for both the unbalanced and cost-sensitive analyses differed considerably (Table 4). The cost-sensitive approach improved the prediction of a breastfeeding duration of <5 months (Table 4).

**Table 4.** Evaluation of Bayesian Network Model

Metrics	Analysis			
	Unbalanced		Cost-sensitive	
	Group A	Group B	Group A	Group B
True positive rate	0.111	0.960	0.405	0.770
False positive rate	0.040	0.889	0.230	0.595
Precision	0.533	0.723	0.420	0.758
Recall	0.111	0.960	0.405	0.770
F-measure	0.184	0.825	0.412	0.764
Receiver operating characteristic area	0.631	0.631	0.631	0.631
Precision–recall curve area	0.413	0.792	0.413	0.792

Breastfeeding duration: Group *A* < 5 months, Group *B* ≥ 5 months.

## Discussion

To our knowledge, this is the first study using data from the ENADID 2018 survey that has demonstrated that determinants of vulnerability, such as lower education, lower sociodemographic stratum, living in a rural area, and considering oneself indigenous, are factors associated with not introducing formula during the first 3 days of life. It is difficult to compare our findings because we did not find studies covering national surveys that investigated the timing of breast milk substitute use. Although the ENADID 2018 survey did not investigate formula supplementation settings or the motives behind formula supplementation, due to the length of hospital stays after birth in Mexico, we hypothesize that most cases may occur in a medical unit.

With the data from the ENADID 2018, it was found that 21% of infants received formula within 3 days of life. In Canada, a study conducted on postpartum units of university teaching hospitals, including 564 mother–baby pairs, reported that 47.9% of infants received formula in the hospital, and the median age at the first supplementation was 8.4 hours.^18^ Planning to exclusively breastfeed, planning to breastfeed for ≥3 months, undergoing childbirth education, completing community college and breastfeeding at delivery have been reported to be “protective against supplementation.”^18^ In the United States, a review of 302 health records found that 114 (38%) healthy newborns were supplemented with formula at the hospital.^19^ In all cases, maternal intention to exclusively breastfeed during the hospital stay was documented. Babies born during the night and early morning were twice as likely to receive supplementation compared with babies born during the day, and the authors suggest that this could be due to maternal fatigue.^19^ A limitation of the ENADID 2018 survey is the lack of questions about maternal breastfeeding intention.

A shorter breastfeeding duration has been associated with in-hospital formula feeding, impacting mother and infant health.^20^ In-hospital supplementation is influenced by a variety of factors, including staff training, mothers' physical and cultural characteristics, hospital policies, and the availability of free formula.^20^ Predictors of breast milk substitute feeding among newborns in delivery facilities in urban Cambodia and Nepal were recommendations from friends/family and cesarean delivery.^21^ The early initiation of breastfeeding and higher parity were “protective against the use of breast milk substitutes.”^21^ Possible indications for supplementation in healthy term infants include hypoglycemia, clinical or laboratory evidence of significant dehydration, hyperbilirubinemia, and weight loss of ≥8–10% on day 5 or later^3^; proper management of lactation can prevent these conditions.^22–24^ Contrary to our expectations, we did not find an association between disability or limitation and introducing formula within the first 3 days of life. It has been reported that mothers with physical impairments perceive breastfeeding as difficult and should be supported.^25,26^

Using machine learning analysis, it has been reported in Brazil that the length of the hospital stay after birth is the most important predictor of feeding practice.^27^ In Mexico, official norms recommend that a patient may be discharged until 24 hours postpartum if there are no complications.^28^ We found that when delivery occurred in a medical unit, most formula supplementation occurred on the first day, and a cesarean section was associated with formula supplementation within the first 3 days of life. The BN analysis showed that the type of delivery, delivery in a medical unit, skin-to-skin contact immediately after birth and receiving an explanation of breastfeeding after delivery are all variables associated with the establishment of breastfeeding.

We did not find similar studies to compare the performance of the metrics generated through BN analysis. A study on lactation in Nigeria showed an accuracy of 97.8% in predicting breastfeeding practices using different machine learning algorithms, but the total number of cases was not included.^29^ Moreover, the authors used a binary classification for breastfeeding practice, normal versus poor, but we did not find the criteria for establishing these categories.^29^ Another study in Spain, which used machine learning algorithms to predict exclusive breastfeeding during postpartum hospital stays, reported an ROC area of 0.78 and a PRC area of 0.86^30^; however, that study was carried out in a regional setting, which included 2,042 cases, and the present study used a dataset from a national survey that included 17,686 cases.

Our analysis showed that the BN model can sufficiently predict cases with a breastfeeding duration of ≥5 months (PRC area = 0.792). In fact, the PRC area has been reported to be more informative than the ROC area using binary classifications in unbalanced databases.^31^ In our analysis, the ROC area did not change in either the unbalanced or cost-sensitive analysis. The PRC area proved to be a robust metric because it provided information on the model's ability to classify cases by group (*A* = 0.413, *B* = 0.792), and the unbalanced data from the ENADID 2018 did not influence the metric.

The strength of this study is the sample design, which was probabilistic and nationally representative and included a considerable number of mother–baby pairs (*N* = 17,686). The results represent support for public health interventions in Mexico to promote breastfeeding. This study has certain limitations due to the methodology and questionnaires used in the ENADID 2018, which use the recall method and are subject to memory bias. Furthermore, the data generated for the ENADID 2018 to measure breastfeeding practices cannot be compared with the breastfeeding indicators proposed by the WHO. The exclusive breastfeeding indicator proposed by the WHO employs the status quo method to determine the prevalence of infants “0–5 months of age who were fed only breast milk during the previous day.”^4^ Other limitations include the lack of survey data on the frequency and intensity of formula introduction. Additionally, the survey did not include a comprehensive analysis of the socioeconomic, cultural, and health care system factors that may influence the introduction of formula and breastfeeding duration, especially breastfeeding in the first hour and rooming-in.

## Conclusions

Focusing on the sensitive window within the first 3 days of a newborn's life, certain lessons can be learned from this study. Maternal attributes of vulnerability, such as less education, low sociodemographic stratum, living in a rural area, and considering oneself indigenous, were factors associated with not introducing formula. For mothers with these socioeconomic conditions, breastfeeding would seem to be the only viable feeding option for their children. It could be speculated that mothers in a higher socioeconomic position may maintain the use of formulas, the cost of which is high. On the other hand, introducing formula within the first 3 days of life was associated with a shorter breastfeeding duration. The BN model revealed a relationship between the type of delivery, clinical practice variables during the immediate postpartum period, and the introduction of formula. Taken together, these results suggest the need to strengthen breastfeeding promotion in clinical settings in Mexico.
